# Book Notices

**Published:** 1866-01

**Authors:** 


### Book Notices.

THE PRACTICE OF MEDICINE. By THOMAS HAWKS TANNER, M.D., F.L.S., Member of the Royal College of Physicians, &c., &c., &c. From the Fifth London Edition; enlarged and improved. Philadelphia: LINDSAY & BLAKISTON. 1866.

This is a new edition of a work which has been long enough before the profession to be well known. The present edition, however, is so much enlarged as to have much the appearance of a new work. It is a full-sized octavo volume of 835 pages, printed on good type and paper, and substantially bound. The author writes in a plain, concise, and agreeable style; and most of his observations in relation to the nature and treatment of diseases, are characterized by good sense and extensive research. As a manual, or text-book, for the use of students, while in attendance at the medical colleges, it would be both convenient and useful, but it is too brief to constitute a satisfactory treatise on the science and practice of medicine. For instance, the consideration of the whole class of idiopathic fevers is compressed into the short space of 42 pages, while more than 100 pages are occupied by an appendix of formulæ. Still the volume is well worth a place in the library of the practitioner.

For sale by W. B. KEEN & Co., 148 Lake Street, Chicago.

---

CHLOROFORM: ITS ACTION AND ADMINISTRATION. By ARTHUR ERNEST SANSOM, M.B., London, late House Physician and Physician-Accoucheur's Assistant to King's College Hospital. Philadelphia: LINDSAY & BLAKISTON. 1866.

This is an American edition of a new English work, on a very interesting and important subject. It is a small-sized octavo volume, containing 279 pages, published in fair style. The work is divided into twenty chapters, with the following titles: I. The Discovery of Chloroform. II. The Influence of the Discovery. III. The Chemistry of Chloroform. IV. The Effects of its Inhalation. V. The Physiological Action of Chloroform and its Allies. VI. The Action of Chloroform and Anæsthetic Agents, &c., upon the Blood and the Circulation—their *modus operandi*. VII. The Danger of Chloroform, and the Circumstances which modify it. VIII. Diseased Conditions which increase the Danger of Chloroform. IX. The Dangers of the Incautious Administration of Chloroform. X. Signs of Danger under the Influence of Chloroform. XI. Nature and Mode of Death from Chloroform. XII. Resuscitation in Apparent Death from Chloroform. XIII. Resuscitation, Practical Details, Summary. XIV. Methods of administering Chloroform. XV. Practical Rules for the Administration of Chloroform. XVI. On Anæsthetic Mixtures. XVII. Chloroform in Surgery. XVIII. Chloroform in Obstetric Practice. XIX. Chloroform in Practical Medicine. XX. Chloroform in Dentistry.

From this table of contents, our readers will readily infer the general scope of the work, as well as the special topics discussed. The author is an enthusiastic advocate of the use of chloroform in preference to other anæsthetic agents. Although, in a hasty perusal, we find many items in reference to which we should dissent from the author's views, yet both students and practitioners will find a careful reading of the work both pleasant and profitable.

For sale by W. B. Keen & Co., 148 Lake St., Chicago.

---

On the Diseases, Injuries, and Malformations of the Rectum and Anus; with Remarks on Habitual Constipation. By T. J. Ashton, formerly Surgeon to the Blenheim Dispensary; Fellow of the Royal Medico-Chirurgical Society; Member of the Pathological Society of London, &c., &c. With Illustrations. Second American, from the Fourth and Revised English Edition. Philadelphia: Henry C. Lea. 1865.

This is a new and carefully revised edition of one of the most valuable special treatises that the physician and surgeon can have in his library. It is a full-sized octavo, of 267 pages. The following important topics are pretty fully and practically considered, viz.:—Irritation and Itching of the Anus; Inflammation and Excoriation of the Anus; Excrescences of the Anal Region; Contraction of the Anus; Fissure of the Anus and Extremity of the Rectum; Inflammation of the Rectum; Ulceration of the Rectum; Hæmorrhoidal Affections; Enlargement of Hæmorrhoidal Veins; Prolapsus of the Rectum; Abscesses near the Rectum; Fistula in Ano; Polypi of the Rectum Stricture of the Rectum; Malignant Diseases of the Rectum; Injuries of the Rectum; Foreign Bodies in the Rectum; Malformations of the Rectum; and Habitual Constipation.

For sale by W. B. KEEN & Co., 148 Lake St., Chicago.

---

THE PRINCIPLES OF SURGERY. By JAMES SYME, F.R.S.E., Surgeon in ordinary to the Queen, in Scotland; Professor of Clinical Surgery in the University of Edinburgh; Member of the General Medical Council, &c., &c., &c. To which are appended his treatises on "The Diseases of the Rectum;" "Stricture of the Urethra, and Fistula in Perinæo;" "the Excision of Diseased Joints," and numerous additional contributions to the pathology and practice of surgery. Edited by his former pupil, DONALD MACLEAN, M.D., L.R.C. S.E., Professor of the Institutes of Medicine, and Lecturer on Clinical Surgery, Queen's University, Canada. Philadelphia: J. B. LIPPINCOTT & Co. 1866.

This is a full-sized octavo volume of 880 pages, published in excellent style. The author is well-known as one of the ablest and most distinguished surgeons in Europe. This treatise on the "Principles of Surgery" occupies the first 524 pages of the present volume. The remaining 356 pages are occupied by an appendix essays on "Diseases of the Rectum;" on "Stricture of the Urethra, and Fistula in Perinæo;" on "Excision of Diseased Joints;" on "Excision of the Scapula and Tongue;" and clinical observations on various surgical topics and cases. The surgical part of the profession, especially, will thank the editor and publishers for giving us this very valuable addition to the list of surgical works.

For sale by S. C. GRIGGS & Co., Lake St., Chicago.

---

ON WAKEFULNESS. With an Introductory Chapter on the Physiology of Sleep. By WILLIAM A. HAMMOND, M.D., Fellow of the College of Physicians of Philadelphia, &c., &c., &c. Philadelphia: J. B. LIPPINCOTT & Co. 1866.

This is a very neatly published little monograph of 93 pages. The substance of it was originally published in the *New York Medical Journal.* It embraces four chapters, viz.: The Physiology of Sleep; the Pathology of Wakefulness; the Exciting Causes of Wakefulness; and the Treatment of Wakefulness.

In regard to the physiology of sleep, the author regards a

*diminution of vascular fulness*, or circulation of blood in the brain, as the immediate or proximate cause of sleep. That sleep and stupor, instead of being analogous conditions, depend on directly opposite states of the cerebral circulation; the first being accompanied by diminished fulness of the cerebral vessels, and the latter by increased fulness.

In regard to the pathology of wakefulness, he says:—"In primary insomnia there is always an increase in the quantity of blood circulating in the brain. This is either absolute or relative."

The following are enumerated by the author, as the exciting causes of wakefulness:—

1st. Long-continued or excessive intellectual action, or any powerful emotion of the mind.

2d. Those positions of the body which tend to impede the flow of blood from the brain, and, at the same time, do not obstruct its passage through the arteries.

3d. An increased determination of blood to the brain, by certain substances used as food or medicine.

4th. Certain functional derangements of such organs as cause an increased accumulation of blood in the brain.

The therapeutic measures recommended by the author, are indicated in the two following propositions:—"1st. Those which by their tendency to soothe the nervous system, or to distract the attention, diminish the action of the heart and bloodvessels, or correct irregularities in their function, and thus lessen the amount of blood in the brain. 2d. Those which directly, either mechanically or through a specific effect upon the circulatory organs, produce a similar effect."

The views of the author, thus briefly indicated, are illustrated in his monograph by the citation of cases and the detail of experiments of a highly interesting and instructive character. We think one of the principal defects in the physiological and pathological views of the author, and, indeed, of most other writers on the same subject, arises from a disposition to attribute all cerebral phenomena to changes in, or differences of, the cerebral circulation. Too exclusive importance is attached to the mere mechanical conditions of greater or less vascular fulness of the brain, while the properties belonging to the brain structure, such as susceptibility and vital affinity, are almost entirely ignored. To this, and some items connected with the author's views of treatment, we may recur hereafter.

The work is for sale by S. C. GRIGGS & Co., Lake Street, Chicago.

---

The following works have been received, and shall be noticed more fully in our next issue, viz.:—

LECTURES ON EPILEPSY, PAIN, PARALYSIS, AND CERTAIN OTHER DISORDERS OF THE NERVOUS SYSTEM. By CHARLES BLAND RADCLIFFE, M.D., Fellow of the Royal College of Physicians of London, &c., &c. Philadelphia: LINDSAY & BLAKISTON. 1866.

RHINOSCOPY AND LARYNGOSCOPY; THEIR VALUE IN PRACTICAL MEDICINE. By Dr. FRIEDERICH SEMELEDER, Physician in ordinary to His Majesty the Emperor of Mexico, Member of the Royal Medical Society of Vienna, &c., &c. Translated from the German, by EDWARD T. CASWELL, M.D. With Illustrations. New York: WILLIAM WOOD & Co., 61 Walker Street. 1866.

